# Editorial: Ethnoveterinary practices in livestock: Animal production, healthcare, and livelihood development

**DOI:** 10.3389/fvets.2022.1086311

**Published:** 2023-01-04

**Authors:** Inayat Ur Rahman, Farhana Ijaz, Rainer W. Bussmann

**Affiliations:** ^1^Department of Botany, Khushal Khan Khattak University, Karak, Khyber Pakhtunkhwa, Pakistan; ^2^Department of Botany, Hazara University, Mansehra, Khyber Pakhtunkhwa, Pakistan; ^3^Department of Ethnobotany, Institute of Botany, Ilia State University, Tbilisi, Georgia; ^4^Department of Botany, State Museum of Natural History, Karlsruhe, Germany

**Keywords:** ethnoveterinary medicine, livestock disease, cattle raising, indigenous knowledge, traditional healthcare system

Ethnoveterinary medicine (EVM) is a field for protecting animal health and treating illnesses that is associated with traditional beliefs and indigenous knowledge and practices (1). Plant-based ethnoveterinary medicine is extensively used across the world since livestock raising is an integral part of most people's livelihoods. Approximately one percent of all known species (about 391,000) has a history of food use, while 10–15% are commonly used in traditional medicine (2). Furthermore, ethnoveterinary medicine is very dynamic and multifunctional since it can cure a variety of livestock illnesses as well as being widely available in remote areas and less expensive than synthetic medicines. Very little attention has been given to documentation of plants used as EVM (3), and this cultural reservoir is disappearing day by day in some parts of the developed world. However, in remote and underdeveloped areas of the world, the EVM is silently playing a vital role in sustainable livestock production. In this editorial, we summarized the main findings and perspectives within each of the accepted articles.

In Namibia's Omusati and Kunene areas, Eiki et al. investigated the ethnoveterinary medicines used to treat livestock diseases. They reported 15 plant species used as veterinary medicines, which were grouped into 10 plant families. Medical formulations usually favor fresh ingredients. The most preferred method of administration was oral, followed by cutaneous (topical). Diarrhea was treated using *Ziziphus mucronata, Combretum collinum*, and *Colophospermum mopane*. Mastitis was treated with *Z. mucronata*. *Salvadora persica* and *Aloe esculenta* were used to cure skin infections. Cattle, goats, and sheep eye diseases were treated with *Ximenia americana* and *C. imberbe*. *Grewia flavescens, Acacia nilotica*, and *A. erioloba* were used to cure retained placentas. *Fockea angustifolia* roots were used to cure anthrax.

In China's Guangxi and Guizhou provinces, the Baiku Yao people were explored by Luo et al. The Baiku Yao are an ethnic group recognized by UNESCO and possess a wealth of ethnoveterinary expertise that they employ to prevent and manage a variety of livestock diseases. The Baiku Yao community's cattle were unharmed by the outbreak of African swine disease. They compiled information on 39 ethnoveterinary plant species that are used to cure a range of illnesses. For curing animal plagues, *Strobilanthes cusia, Tetradium ruticarpum*, and *Stephania kwangsiensis* are particularly valued locally.

Domestic animals play a key role in the evolution of human civilization. In this regard, plants are used to treat a range of livestock. The tribal populations of North Waziristan, Khyber Pakhtunkhwa, Pakistan were the subject of an investigation by Rehman et al. because they were quite knowledgeable about the therapeutic potential of medicinal plants as ethnoveterinary remedies. They noted the usage of 56 medicinal plant species from 42 families to treat 45 distinct animal illnesses. Treatments for blood in the urine, bone damage, colic, indigestion, postpartum retention, skin disorders, constipation, increased milk production, mastitis, foot, and mouth ailments were among the most frequently reported ethnoveterinary uses.

The ethnoveterinary and ethnopharmacological practices were examined by Rivera et al. in the context of the historic transhumance routes that cross Castilla La Mancha from north to south. The interviews were performed between 1994 and 2021 in 86 communities along eight major transhumance routes, or “caadas reales,” and 25 other minor transhumance routes. There is consideration of 63 disorders that have been recorded with their remedies. From the interviews, 202 plant species belonging to 92 families have been documented. In the interviews, *Erophaca baetica, Lupinus angustifolius*, and *Oenanthe crocata* were the three poisonous plant species that were most frequently mentioned. Some of the species listed as poisonous were disease reservoirs or indicators of hazardous regions. The research's coverage of endo-and ectoparasite prevention, protection, and control was one of its most comprehensive subjects of study.

According to Cáceres et al., obtaining veterinary care and developing a thorough understanding of conventional ethnoveterinary practices are essential for the welfare of animals. This work aims to contribute to this issue in the Catalan linguistic area by concentrating on the study of plants used to treat and deal with gastrointestinal, metabolic, and nutritional disorders in livestock. The expertise of 599 informants, who together provided 1,405 reports of use from 148 plant species, was used to gather their expertise on plants used in veterinary medicine. 95.02% of all use reports refer to digestive system illnesses, 4.34% to nutritional disorders, and 0.64% to metabolic disorders. Tisane was the most common method of preparation and administration (58.69%). The findings of this study are pertinent to the documentation of ethnoveterinary knowledge and provide a significant contribution to the study of potential novel plant-based livestock medications.

According to Uprety et al., traditional herbal treatments are practiced all over the world to address both human and animal health problems. The usage of 393 plant species from 114 families in ethnoveterinary practices is reported from Nepal. This study demonstrates that traditional herbal therapy is a legitimate practice and plays a key role in addressing the livestock healthcare needs of Nepali farmers. This could also give livestock producers a choice if they cannot afford allopathic treatment or if they are prohibited from using it by organic farming practices, both of which are likely to be included in Nepal's future sustainable livestock farming initiatives.

As per Haq et al., ethnic knowledge is deeply ingrained in rural communities and is strongly connected to the local biodiversity of Jammu and Kashmir Himalayan regions. In this context, they interviewed the study area local inhabitants and reported 148 species from 53 families as medicine, food, tonics, antidotes, magic, and as defenses against ectoparasites. *Amaranthus blitum, Morus alba, Ficus palmata, Vitex negundo, Juniperus semiglobosa, Ulmus wallichiana*, and *Rumex nepalensis* have not before been reported for their current usage in this studied region of the Himalaya. The novelty of the current study can be attributed to the several unique fodder preparations (gaas lov, gass khor, pan baath, kaand baath, Lov gooad, Karb, Phungma) from indigenous plants that are documented for the first time in the Himalayan region.

## Author contributions

All authors listed have made a substantial, direct, and intellectual contribution to the work and approved it for publication.
